# Lipocalin-2 promotes NSCLC progression by activating the JAK2/STAT3 signaling pathway

**DOI:** 10.1186/s12967-025-06418-1

**Published:** 2025-04-10

**Authors:** Jinjin Zhang, Qin Xu, Gengyun Sun

**Affiliations:** Department of Respiratory and Critical Care Medicine, The First Affiliated Hospital of Anhui Medical University, Anhui Province 230022 Hefei, China

**Keywords:** Lipocalin-2, Non-small cell lung cancer, JAK2/STAT3 signaling pathway, Progression, Metastasis

## Abstract

**Background:**

Non-small cell lung cancer (NSCLC) remains a leading cause of cancer-related mortality worldwide. Lipocalin-2 (LCN2), a pleiotropic protein implicated in tumorigenesis and cancer progression, has been associated with multiple malignancies. However, its precise role in NSCLC and the underlying molecular mechanisms remain incompletely understood. This study aimed to elucidate the function of LCN2 in NSCLC, with a particular focus on its involvement in the Janus kinase 2/signal transducer and activator of transcription 3 (JAK2/STAT3) signaling pathway.

**Methods:**

LCN2 expression in NSCLC tissues was comprehensively analyzed using bioinformatics tools, including the Universal Analysis of Cancer (UALCAN), The Cancer Genome Atlas (TCGA), UCSC-XENA, and Gene Expression Omnibus (GEO) databases. Quantitative real-time polymerase chain reaction (qRT-PCR) and western blotting were employed to assess LCN2 expression levels in NSCLC cell lines. The functional impact of LCN2 on NSCLC cells, including proliferation, apoptosis, and metastasis, were assessed through a series of in vitro assays, such as Cell Counting Kit-8 (CCK-8), EdU, wound healing, and transwell migration and invasion assays. An in vivo xenograft model was established to investigate the effects of LCN2 on tumor growth and metastasis. Additionally, the involvement of the JAK2/STAT3 signaling pathway was examined using western blotting and pharmacological inhibition with AG490.

**Results:**

LCN2 was significantly upregulated in NSCLC tissues and cell lines, and its elevated expression correlated with poor prognosis. Functional analyses demonstrated that LCN2 knockdown suppressed NSCLC cell proliferation, migration, and invasion while promoting apoptosis. Mechanistically, LCN2 was found to activate the JAK2/STAT3 pathway by interacting with SOCS3, and pharmacological blockade of this pathway effectively abrogated the oncogenic effects of LCN2 overexpression.

**Conclusions:**

This study identifies LCN2 as a potential oncogene in NSCLC, driving tumor progression through activation of the JAK2/STAT3 signaling pathway. These findings suggest that targeting LCN2 or its downstream signaling components may represent a promising therapeutic strategy for NSCLC.

**Supplementary Information:**

The online version contains supplementary material available at 10.1186/s12967-025-06418-1.

## Background

Lung cancer remains a leading cause of cancer-related mortality worldwide, characterized by high incidence rates and poor survival outcomes. Non-small cell lung cancer (NSCLC), the predominant histological subtype, accounts for over 85% of all lung cancer cases [1]. NSCLC is an aggressive malignancy that is often diagnosed at advanced stages with local or distant metastases, significantly limiting therapeutic options and leading to a poor prognosis [2]. Despite considerable advancements in cancer research and the development of innovative treatment strategies, the 5-year relative survival rate for NSCLC patients remains dismally low [3]. Therefore, identifying novel biomarkers and therapeutic targets, as well as elucidating their underlying mechanisms, is crucial for improving NSCLC treatment.

Lipocalin-2 (LCN2), also known as neutrophil gelatinase-associated lipocalin, siderocalin, or oncogene 24p3, is a well-characterized secreted glycoprotein with multifunctional roles in diverse biological processes. This protein is widely expressed across various tissues and cell types and is implicated in numerous physiological and pathological conditions, including inflammation, innate immunity, tissue injury, and cancer progression [4–7]. However, the role of LCN2 in tumorigenesis remains controversial, as it can function as either an oncogene or a tumor suppressor depending on the cancer type. In malignancies such as ovarian cancer [8], breast cancer [9], pancreatic cancer [10], and oral squamous cell carcinoma [11], LCN2 has been identified as an oncogene that promotes tumor progression and metastasis. Conversely, in hepatocellular carcinoma [12] and gastric cancer [13], LCN2 appears to exert tumor-suppressive effects, suggesting a context-dependent role in cancer development. In NSCLC, the functional implications of LCN2 remain incompletely understood. Some studies have suggested that LCN2 promotes ferroptosis and tissue atrophy in mouse models of lung cancer, thereby contributing to cachexia symptoms [14]. In contrast, findings by Casey T. Finnicum et al. indicate that LCN2 may inhibit the progression of tobacco-associated NSCLC [15]. Given these conflicting reports, further investigation is necessary to clarify the precise role of LCN2 in NSCLC pathogenesis.

The Janus kinase (JAK) family, comprising four non-receptor tyrosine kinases (JAK1, JAK2, JAK3, and TYK2), serves as a critical mediator of the signal transducer and activator of transcription (STAT) proteins, initiating phosphorylation events at specific tyrosine residues [16]. Among these, JAK2 activation leads to the phosphorylation of STAT3, which subsequently dimerizes and translocates to the nucleus to regulate the progression of various malignancies, including hepatocellular carcinoma [17], NSCLC [18], and osteosarcoma [19]. As a member of the suppressor of cytokine signalling (SOCS) family, SOCS3 is an important regulator of the JAK2/STAT3 pathway, inhibiting JAK2 activation through multiple mechanisms, thereby reducing STAT3 phosphorylation and preventing its nuclear translocation to modulate gene expression [20, 21]. Increasing evidence highlights the pivotal role of the JAK2/STAT3 signaling pathway in the pathogenesis of multiple cancers, where it regulates essential biological processes such as cell proliferation, apoptosis, and metastasis [17, 22, 23]. However, the precise role of JAK2/STAT3 signaling in NSCLC mediated by LCN2 remains largely unexplored.

In this study, we conducted a comprehensive analysis of multiple NSCLC cohorts and identified a significant upregulation of LCN2 expression, which correlated with poor prognosis. Functional assays demonstrated that LCN2 depletion suppressed both the proliferative and metastatic capacities of NSCLC cells in vitro and in vivo. More importantly, mechanistic investigations revealed that LCN2 promotes activation of the JAK2/STAT3 pathway by directly interacting with SOCS3. These findings provide novel insights into the oncogenic role of LCN2 in NSCLC and establish a potential foundation for future targeted therapeutic strategies.

## Materials and methods

### Patient samples

Paraffin-embedded samples of carcinomas and paired adjacent tissues were obtained from 44 patients with histopathologically confirmed NSCLC who were hospitalized between August 2021 and October 2022 at the First Affiliated Hospital of Anhui Medical University. None of the patients had received prior radiotherapy or chemotherapy. The study protocol was approved by the Ethics Committee of Clinical Medical Research.

### Cell lines, cell culture, and transfection

Human bronchial epithelial cells (Beas-2b) and four NSCLC cell lines (A549, H1299, H1975, and PC9) were obtained from the Cell Bank of the Chinese Academy of Sciences (Shanghai, China). Beas-2b and A549 cells were cultured in DMEM medium (HyClone, USA), while H1299, H1975, and PC9 cells were cultured in RPMI-1640 medium (HyClone), both supplemented with 10% fetal bovine serum (FBS; Gibco, USA) and 1% antibiotics (penicillin/streptomycin; Gibco) at 37 ℃ with 5% CO_2_.

Lentiviral vectors for small hairpin RNA (shRNA) targeting LCN2 and a control vector were constructed using the LV3-CMV-GFP-puromycin system (GenePharma, Shanghai, China). For stable LCN2 knockdown, cells were infected with lentivirus at an MOI of 10 and selected with 2 µg/mL puromycin for 2 weeks. The selected cell lines were maintained in medium containing 1 µg/mL puromycin. Overexpression plasmids pcDNA3.1-LCN2 and pcDNA3.1-LCN2-Flag were constructed using LCN2 cDNA (GenePharma). Using full-length LCN2 cDNA as a template, the pcDNA3.1-LCN2-Mut-Flag plasmid was generated by deleting the interaction site between LCN2 and SOCS3 proteins (amino acids 136–178). Transfections were performed using Lipofectamine 3000 (Invitrogen, USA). Briefly, 2.5 µg of plasmid DNA was diluted in 125 µL Opti-MEM with 5 µL p3000 and 5 µL lipo3000, mixed, and incubated at room temperature for 15 min before addition to 6-well plates. Cells were collected 48–72 h post-transfection for analysis. The shRNA sequence targeting LCN2 was sh-LCN2#3: 5’-GAGCTGACTTCGGAACTAA-3’.

### Co-immunoprecipitation (Co-IP)

Total cell lysates were prepared using IP lysis buffer (Beyotime, China) and incubated with specific primary antibodies overnight at 4 ℃. Protein A/G agarose (Santa Cruz Biotechnology, USA) was added and incubated for 2 h at room temperature. Immune complexes were washed and analyzed by western blotting.

### Western blotting

Total cellular proteins were isolated using RIPA buffer supplemented with PMSF (Beyotime, China) and phosphatase inhibitors (Solarbio, China). Protein concentrations were quantified using a BCA protein assay kit (Epizyme, China). Proteins were separated by SDS-PAGE, transferred to PVDF membranes (Millipore, USA), and blocked with 5% skim milk in TBST for 2 h at room temperature. Membranes were incubated with primary antibodies against LCN2 (1:1000, Huabio, ET1703-39), p-JAK2 (1:1000, Abmart, T56570), JAK2 (1:1000, CST, #3230), p-STAT3 (1:1000, Abmart, T56566), STAT3 (1:1000, CST, #9139), SOCS3 (1:2000, Proteintech, 14025-1-AP), E-cadherin (1:1000, CST, #14472), Vimentin (1:1000, Abcam, ab92547), Bcl2 (1:1000, Abcam, ab32124), BAX (1:1000, Affinity, AF0120), and β-actin (1:10,000, Huabio, EM21002) overnight at 4 ℃. Membranes were then incubated with HRP-conjugated secondary antibodies for 1 h at room temperature, and protein bands were detected using an enhanced chemiluminescence system (Epizyme) and imaged with a Tanon-5200 system (Tanon Science, China).

### Quantitative real-time polymerase chain reaction (qRT‒PCR)

Total RNA was isolated using TRIzol reagent (Invitrogen, USA) and reverse-transcribed into cDNA using Evo M-MLV reverse transcriptase (Accurate Biotechnology, China). qRT‒PCR was performed on a LightCycler^®^ 480 system (Roche, Switzerland) using an qRT-PCR kit (Accurate Biotechnology, China). Gene expression levels were normalized to GAPDH using the 2^^−ΔΔCt^ method. Primers used were: LCN2 forward: 5’-GTGAGCACCAACTACAACCAGC-3’, reverse: 5’-GTTCCGAAGTCAGCTCCTTGGT-3’; GAPDH forward: 5’-AGCCACATCGCTCAGACAC-3’, reverse: 5’-GCCCAATACGACCAAATCC-3’.

### Immunohistochemistry (IHC)

Formalin-fixed, paraffin-embedded lung tissue sections were dewaxed, rehydrated, and subjected to antigen retrieval using citrate buffer (10 mM, pH 6.0). Sections were blocked with 3% hydrogen peroxide and 10% goat serum, then incubated with primary antibodies against LCN2 (1:200, Huabio, ET1703-39) overnight at 4 ℃. After incubation with secondary antibodies for 1 h at room temperature, sections were visualized with diaminobenzidine (DAB) and counterstained with hematoxylin. LCN2 expression was evaluated by at least two blinded pathologists.

### Cell proliferation assays

Transfected cells were seeded into 96-well plates at 1 × 10^3^ cells per well. At 0, 24, 48, 72, and 96 h, 10 µL of Cell Counting Kit-8 (CCK-8, Goonie, China) solution was added to each well and incubated at 37 ℃ for 2 h. Subsequently, the absorbance was measured at 450 nm using a microplate reader (PerkinElmer, USA).

### 5-ethynyl-2’-deoxyuridine (EdU) proliferation assay

The EdU proliferation assay was performed according to the manufacturer’s protocol (Beyotime, China). Briefly, transfected cells were preseeded in 48-well plates and cultured for 12 h, followed by labeling with 10 µM EdU for 2 h. After fixation with 4% formaldehyde and permeabilization with Triton X-100, nuclei were stained with Hoechst 33,342. Fluorescence imaging was performed using a microscope (Zeiss, Germany), and the EdU positive rate was calculated as the ratio of EdU-positive to Hoechst-positive cells.

### Wound healing assay

Transfected cells were seeded into 6-well plates to form a complete monolayer. Once the cells reached approximately 90% confluence, wounds were introduced by scraping the monolayer with a 200 µL pipette tip. After washing with PBS, cells were cultured in low-serum medium (1%). Wound healing was imaged at specified time points and quantified using ImageJ software.

### Transwell assay

Transwell assays for cell migration and invasion were performed using 8.0 μm pore polycarbonate membranes (Falcon, USA). For invasion assays, 1 × 10^5^ transfected cells in serum-free medium were plated onto the upper chamber coated with Matrigel (BD, USA), while for migration assays, cells were plated without Matrigel. The lower chamber contained 10% FBS medium. After incubation for 12–48 h, cells on the upper surface were removed, whereas cells on the lower surface were fixed with 4% paraformaldehyde and stained with 0.1% crystal violet. Random fields were counted and photographed using a microscope (Zeiss, Germany).

### Cell apoptosis analysis

Cell apoptosis was assessed using Annexin V-Alexa Fluor 647/PI staining (Yeasen, China). Cells were collected at 70-80% confluence, resuspended in cold PBS, stained with 5 µL Annexin V-Alexa Fluor 647 and 10 µL PI for 15 min at 4 ℃ in the dark. Apoptotic cells were quantified by flow cytometry (Beckman, USA).

### Animals and animal models

Female BALB/c nude mice (5–6 weeks old) were purchased from Skbex Biotechnology Co., Ltd. (Henan, China) and maintained under specific pathogen-free (SPF) conditions. All animal protocols were approved by the Animal Ethics Committee of Anhui Medical University.

For tumor xenograft studies, A549 cells stably transfected with control shRNA or sh-LCN2 (5 × 10^6^ cells) were injected into the right axillary region of nude mice (*n* = 6 per group). Tumor volume was measured every 3 days using the formula: volume = (length × width^2^ × π)/6. Thirty days post-injection, mice were sacrificed, and tumors were excised, weighed, and photographed. Tumor samples were fixed in 4% paraformaldehyde and subjected to IHC staining using antibodies against LCN2 (1:200, Proteintech, 30576-1-AP), E-cadherin (1:100, CST, #14472), Vimentin (1:200, Abcam, ab92547), and Ki-67 (1:500, Proteintech, 27309-1-AP).

For the lung metastasis model, A549 cells stably transfected with control shRNA or sh-LCN2 (2 × 10^6^ cells) were injected intravenously into the tail vein of nude mice (*n* = 6 per group). Eight weeks later, mice were sacrificed, and lungs were resected, photographed, and fixed in 4% paraformaldehyde for further analysis.

### Hematoxylin–eosin staining (H&E)

Slides were prepared following standard IHC protocols, including deparaffinization and rehydration. Tissue sections were stained with hematoxylin and eosin, dehydrated with graded alcohols, cleared in xylene, and mounted.

### TUNEL staining assay

TUNEL staining was performed using the TUNEL Apoptosis Assay kit (Yeasen, China) according to the manufacturer’s instructions. Tissue slides were incubated in the TUNEL reaction mixture for 1 h at room temperature, followed by staining with DAPI for 5 min. Samples were imaged using a fluorescence microscope (Leica, Germany).

### Statistical analysis

Statistical analyses were performed using SPSS 22.0 (SPSS Software, USA) and GraphPad Prism 9.0 (GraphPad Software, USA). Data were presented as means ± SEM and were analyzed using Student’s t-tests, multiple t-tests, one-way ANOVA, or two-way ANOVA. *P*-values < 0.05 were considered significant (*P < 0.05; **P < 0.01; ***P < 0.001).

## Results

### LCN2 is overexpressed and predicts unfavorable prognosis in NSCLC

**Fig. 1:**
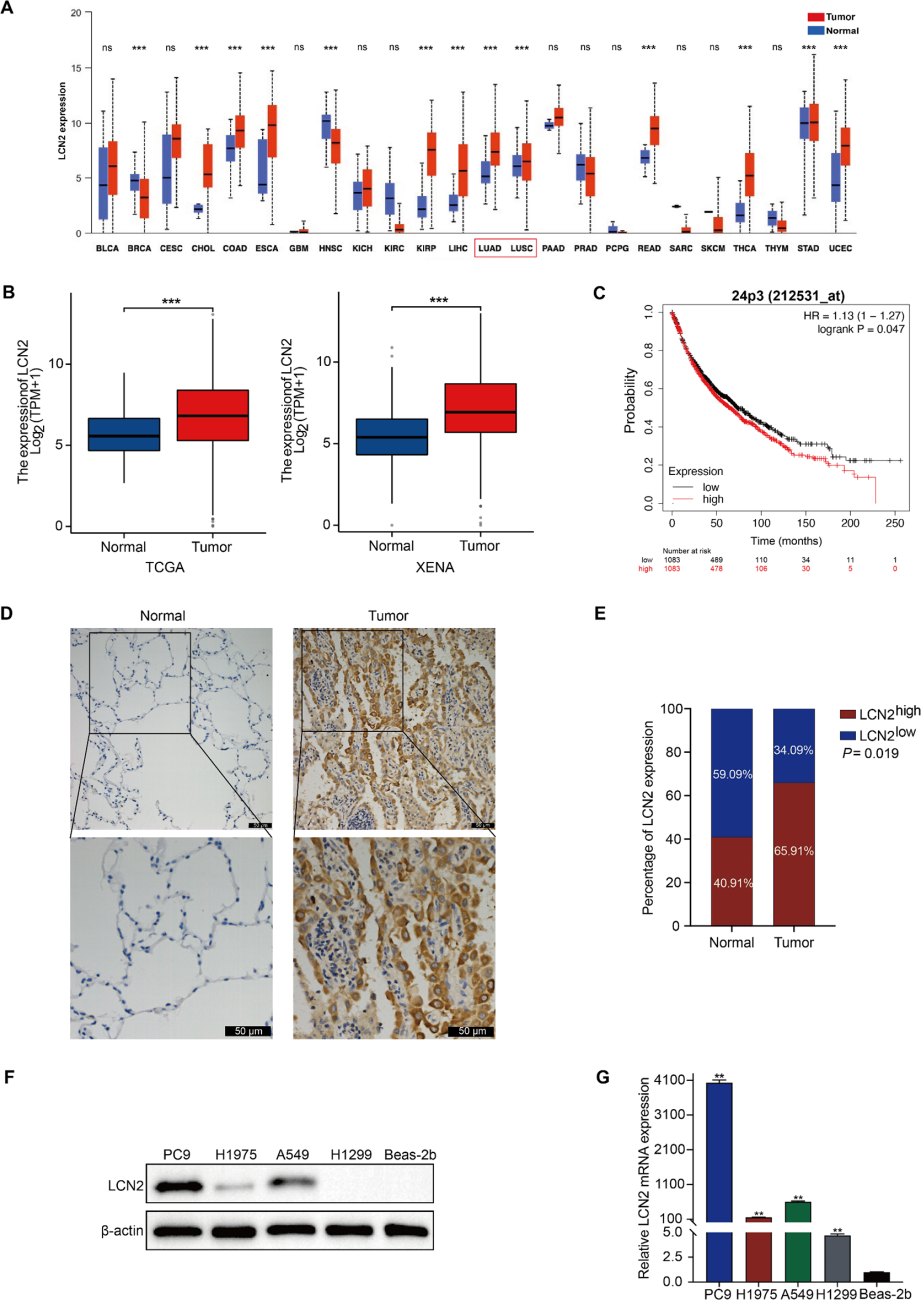
Upregulation of LCN2 in NSCLC and its association with prognosis. (**A**) LCN2 expression across various cancer types was analyzed using the UALCAN database (https://ualcan.path.uab.edu). (**B**) LCN2 expression levels in NSCLC were examined using TCGA (left) (https://portal.gdc.cancer.gov) and UCSC-XENA (right) (https://xena.ucsc.edu) databases. (**C**) Kaplan-Meier survival analysis of OS based on LCN2 expression in NSCLC was obtained from the KM plotter database (https://kmplot.com). (**D**) Representative IHC staining images showing LCN2 expression in NSCLC patient specimens. (**E**) Statistical analysis of LCN2 expression in NSCLC and adjacent non-cancerous tissues. (**F**, **G**) Western blot and qRT-PCR analyses the relative LCN2 expression levels in BEAS-2B and NSCLC cell lines. Data are presented as mean ± SEM (*n* = 3). *P < 0.05; **P < 0.01; ***P < 0.001

We initially examined LCN2 mRNA expression across various cancers using the Universal Analysis of Cancer (UALCAN) database (https://ualcan.path.uab.edu), revealing elevated LCN2 levels in tumor tissues compared to normal tissues in lung adenocarcinoma (LUAD) and lung squamous cell carcinoma (LUSC) (Fig. 1A). We further assessed LCN2 mRNA expression in human NSCLC using data from The Cancer Genome Atlas (TCGA) (https://portal.gdc.cancer.gov), UCSC XENA (https://xena.ucsc.edu), and Gene Expression Omnibus (GEO) (https://www.ncbi.nlm.nih.gov/geo/) databases. Our analyses consistently showed significantly higher LCN2 expression in tumor tissues compared to normal tissues (*P* < 0.001, Fig. 1B, and Fig. S1A). Kaplan-Meier survival analysis using the KM Plotter (https://kmplot.com) and the GuangRe biotechnology database (https://grswsci.top) demonstrated that elevated LCN2 expression was associated with reduced overall survival (OS) in NSCLC patients (Fig. 1C, and Fig. S1B-D). IHC analysis confirmed higher LCN2 expression in NSCLC tissues compared to adjacent non-tumorous tissues (*P* = 0.019, Fig. 1D & E). Additionally, we correlated LCN2 expression with clinicopathological parameters and found a significant association with tumor N stage (*P* = 0.038, Table 1). We also constructed a nomogram model incorporating LCN2 expression and clinical prognostic parameters in a large-sample META cohort (GSE31210 + GSE72094), which showed good predictive accuracy for 1-, 3-, and 5-year OS (Fig. S1E). The calibration curve proves that the accuracy of the model is consistent with the actual situation (Fig. S1F). Decision curve analysis indicated that the nomogram model outperformed other prognostic indicators (Fig. S1G), and AUC values further confirmed its superior accuracy in predicting OS (Fig. S1H). Moreover, LCN2 expression was elevated in NSCLC cell lines (A549, H1299, H1975, and PC9) compared to the non-malignant Beas-2b cell line (Fig. 1F & G). Collectively, these findings indicate that LCN2 is upregulated in NSCLC and may serve as a valuable prognostic biomarker for OS.

**Table 1:** Correlation between LCN2 expression and clinicopathologic parameters in NSCLC patients. *P < 0.05

	Variables	LCN2 expression		Total	χ 2	*P* value
		high	low			
Sex					4.130	0.042*
	Female	10	10	20		
	male	19	5	24		
Age (year)					3.334	0.068
	< 65	14	3	17		
	≥ 65	15	12	27		
T stage					2.276	0.131
	T1/T2	25	15	40		
	T3/ T4	4	0	4		
N stage					4.306	0.038*
	N0/Nx	22	15	37		
	N1/N2/N3	7	0	7		
M stage					0.529	0.467
	M0	28	15	43		
	M1	1	0	1		
TNM stage					2.918	0.088
	I/ II	24	15	39		
	III/IV	5	0	5		

*P < 0.05

### LCN2 promotes NSCLC proliferation

**Fig. 2:**
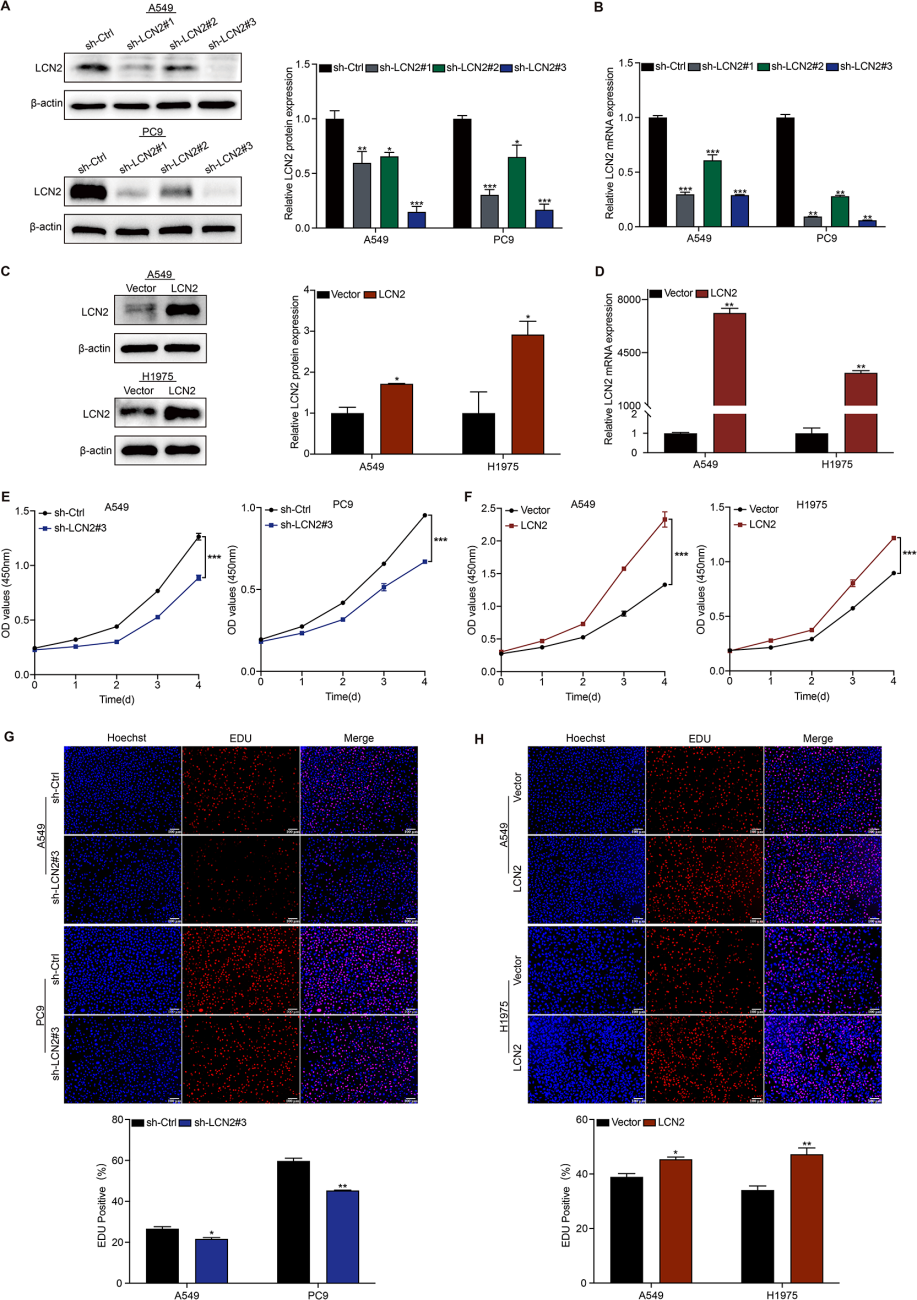
LCN2 promotes NSCLC proliferation in vitro. (**A**, **B**) Western blot and qRT-PCR validation of LCN2 knockdown efficiency in A549 and PC9 cells. (**C**, **D**) Western blot and qRT-PCR confirmation of LCN2 overexpression efficiency in A549 and H1975 cells. (**E**, **F**) CCK-8 assay assessing the proliferation of NSCLC cells following LCN2 knockdown or overexpression. (**G**, **H**) EdU assay evaluating the effects of LCN2 knockdown or overexpression on NSCLC cell proliferation. Data are presented as mean ± SEM (*n* = 3). *P < 0.05; **P < 0.01; ***P < 0.001

A series of functional assays were performed to elucidate the role of LCN2 in NSCLC. We selected A549 and PC9 cell lines for LCN2 knockdown due to their high endogenous LCN2 expression, while A549 and H1975 cell lines were chosen for overexpression studies. Among three shRNAs targeting LCN2, shLCN2#3 showed the most pronounced silencing effect, validated by qRT-PCR and western blot analysis (Fig. 2A & B). Similarly, LCN2 overexpression efficiency was confirmed in these cell lines (Fig. 2C & D). The CCK-8 assay revealed that LCN2 knockdown significantly suppressed cell proliferation (Fig. 2E), whereas LCN2 overexpression promoted it (Fig. 2F). These findings were corroborated by EdU assays, which showed decreased proliferation upon LCN2 silencing (Fig. 2G) and increased proliferation with LCN2 overexpression (Fig. 2H). Collectively, these data suggest that LCN2 promotes NSCLC cell proliferation in vitro.

### LCN2 facilitates NSCLC metastasis

**Fig. 3:**
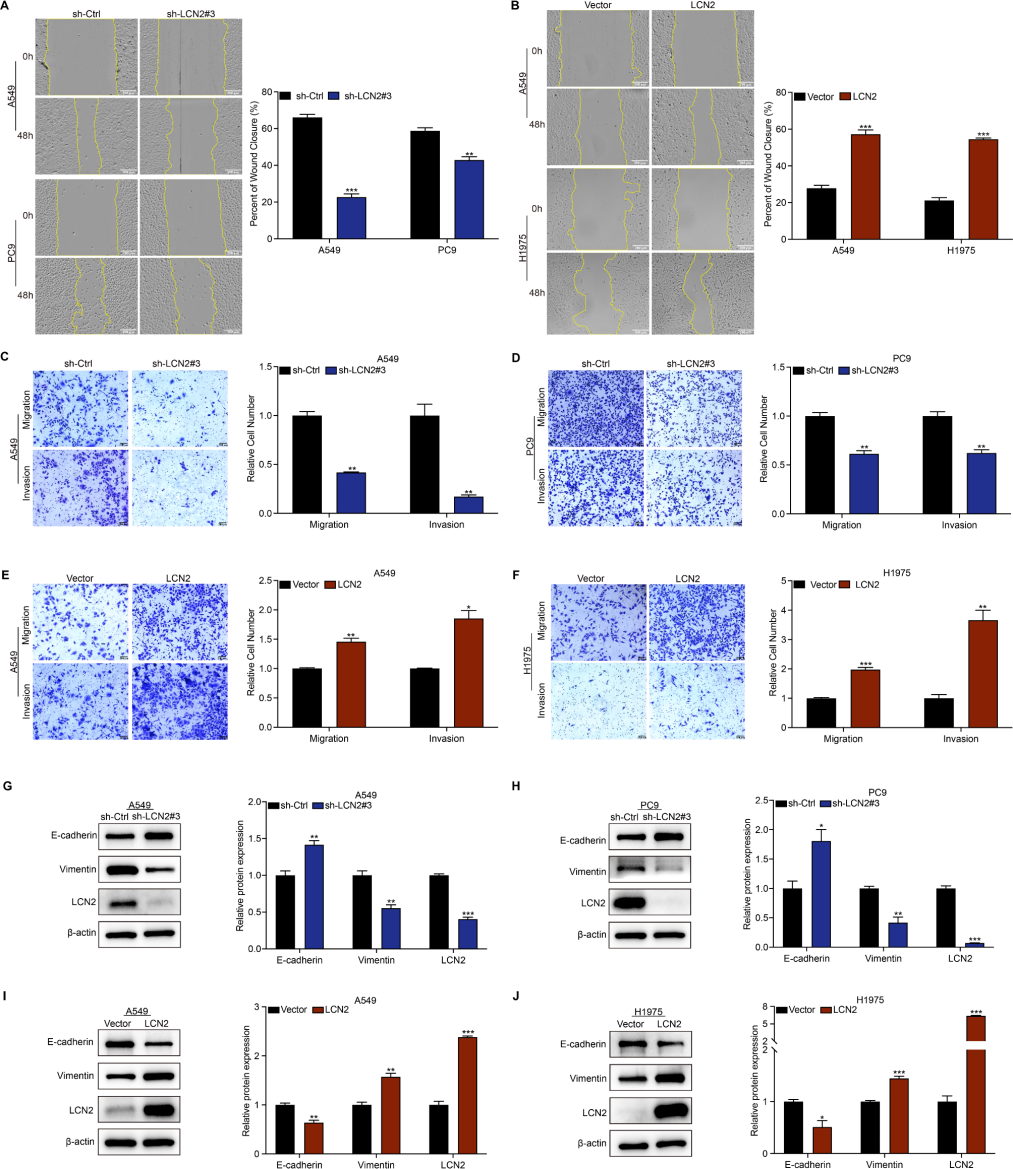
LCN2 enhances NSCLC metastasis in vitro. (**A**, **B**) Wound healing assay assessing the migratory capacity of NSCLC cells following LCN2 knockdown or overexpression. (**C**-**F**) Transwell migration and invasion assays evaluating the metastatic potential of NSCLC cells with altered LCN2 expression. (**G**-**J**) Western blot analysis of E-cadherin and Vimentin expression levels. Data are presented as mean ± SEM (*n* = 3). *P < 0.05; **P < 0.01; ***P < 0.001

We further investigated the role of LCN2 in NSCLC metastasis using wound healing and transwell assays. Wound healing assays showed that LCN2 knockdown significantly attenuated cell migration (Fig. 3A), while LCN2 overexpression enhanced it (Fig. 3B). Consistently, transwell assays demonstrated that LCN2 knockdown reduced cell migration and invasion through an extracellular matrix barrier (Fig. 3C & D), whereas LCN2 overexpression increased these capabilities (Fig. 3E & F).

Given the involvement of epithelial-mesenchymal transition (EMT) in cancer metastasis, we examined EMT markers (E-cadherin and Vimentin) via western blotting. LCN2 knockdown upregulated E-cadherin and downregulated Vimentin (Fig. 3G & H), while LCN2 overexpression had the opposite effect (Fig. 3I & J). These results suggest that LCN2 facilitates NSCLC cell invasion and migration by modulating EMT.

### LCN2 suppresses apoptosis in NSCLC

**Fig. 4:**
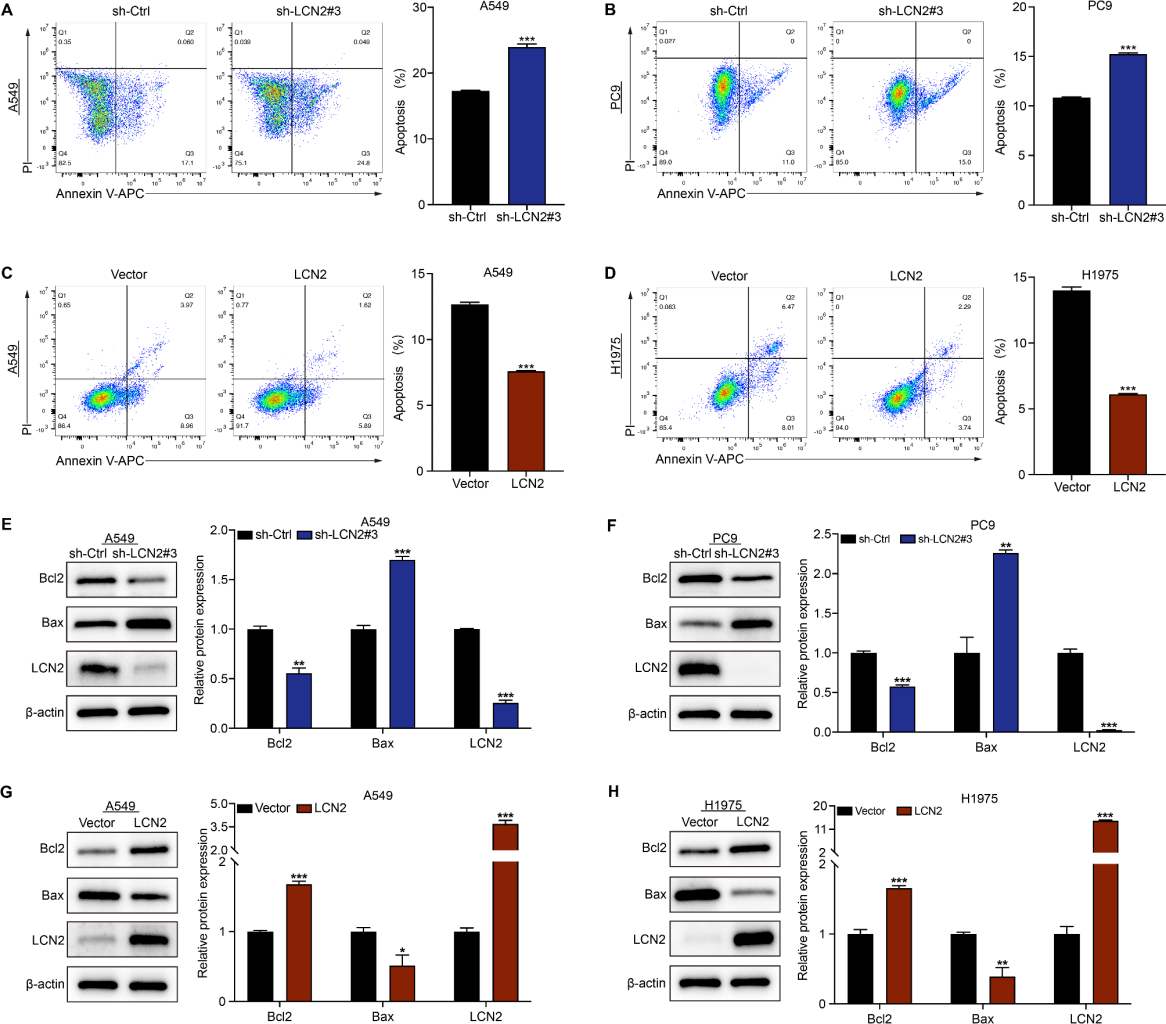
LCN2 inhibits apoptosis in NSCLC cells. (**A**, **B**) Flow cytometry analysis of apoptosis in A549 and PC9 cells following LCN2 knockdown. (**C**, **D**) Flow cytometry analysis of apoptosis in A549 and H1975 cells following LCN2 overexpression. (**E**-**H**) Western blot analysis of apoptotic markers Bax and Bcl2. Data are presented as mean ± SEM (*n* = 3). *P < 0.05; **P < 0.01; ***P < 0.001

We also explored the role of LCN2 in modulating apoptosis in NSCLC cells. Flow cytometry analysis revealed an inverse correlation between LCN2 levels and apoptosis incidence (Fig. 4A-D). Western blot analysis showed that LCN2 knockdown decreased the anti-apoptotic protein Bcl-2 levels and increased the pro-apoptotic protein Bax expression (Fig. 4E & F), while LCN2 overexpression had the opposite effect (Fig. 4G & H). Collectively, these findings indicate that LCN2 protects NSCLC cells from apoptosis in vitro.

### LCN2 knockdown reduces NSCLC tumor growth and metastasis in vivo

**Fig. 5:**
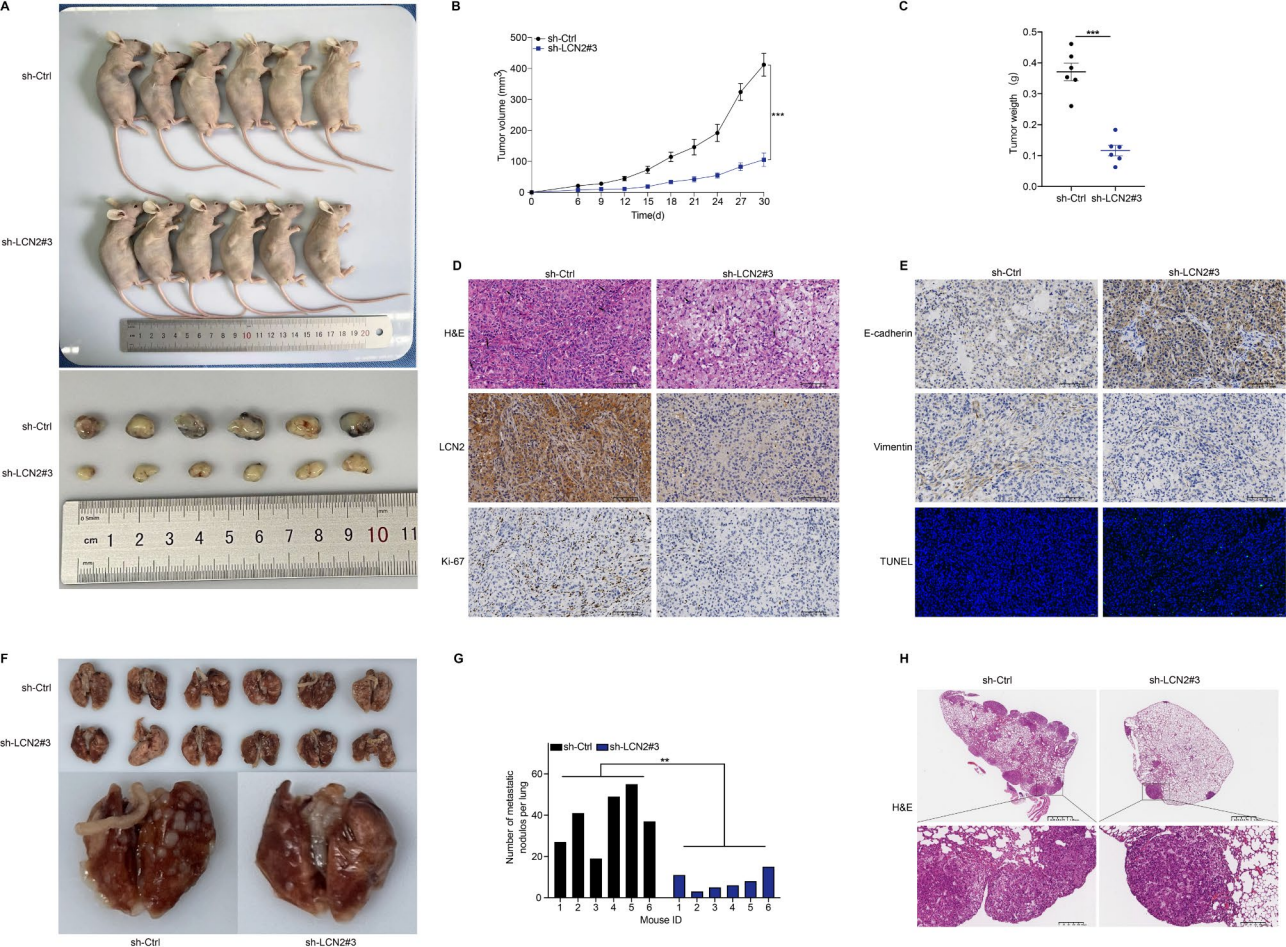
LCN2 knockdown suppresses tumor growth and metastasis in vivo. (**A**) Representative images of nude mice and subcutaneous xenograft tumors derived from LCN2-knockdown and control A549 cells. (**B**, **C**) Tumor volume and weight comparisons between the two groups. (**D**, **E**) Representative H&E, TUNEL, and IHC staining images of LCN2, Ki67, E-cadherin, and Vimentin expression in xenograft tumor tissues. (**F**) Lung specimens from a metastatic NSCLC mouse model. (**G**) Quantification of lung metastases in LCN2-knockdown and control groups. (**H**) Representative H&E-stained images of lung lesions in the metastasis model. Data are presented as mean ± SEM (*n* = 6). *P < 0.05; **P < 0.01; ***P < 0.001

Mouse models were utilized to further elucidate the oncogenic role of LCN2 in NSCLC. In a subcutaneous xenograft model, tumors derived from sh-LCN2 A549 cells exhibited significantly reduced weight and volume compared to those from sh-Ctrl cells (Fig. 5A-C). H&E staining revealed a reduction in pathological mitotic figures in the sh-LCN2 group compared to the sh-Ctrl group (Fig. 5D). Additionally, IHC analysis showed decreased expression of LCN2 and Ki67, increased expression of E-cadherin, and reduced levels of Vimentin in the sh-LCN2 group relative to the sh-Ctrl group (Fig. 5D & E). TUNEL staining indicated increased apoptosis in the sh-LCN2 group (Fig. 5E).

In a tail vein injection model, pulmonary metastatic foci were significantly reduced in the LCN2 knockdown group compared to controls (Fig. 5F & G). H&E staining confirmed extensive pulmonary metastasis in the sh-Ctrl group, while the sh-LCN2 group exhibited fewer metastatic lesions (Fig. 5H). These results demonstrate that LCN2 knockdown inhibits NSCLC growth and metastasis in vivo.

### LCN2 regulates NSCLC progression via the JAK2/STAT3 signaling pathway

**Fig. 6:**
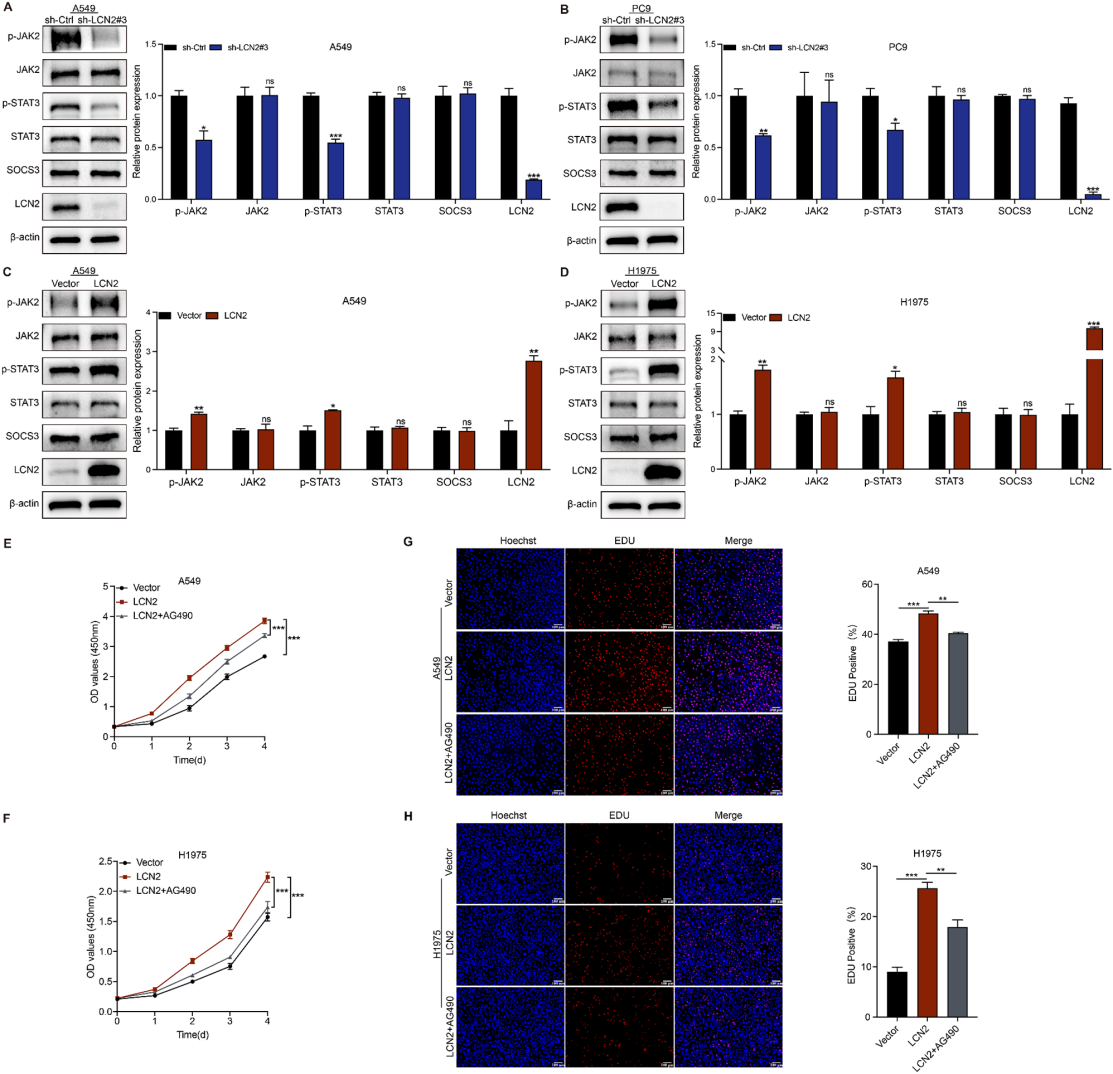
LCN2 promotes NSCLC proliferation via the JAK2/STAT3 pathway. (**A**-**D**) Western blot analysis of p-JAK2, JAK2, p-STAT3, STAT3, SOCS3, and LCN2 expression in NSCLC cells. (**E**, **F**) CCK-8 assay evaluating the effect of AG490 treatment on LCN2-overexpressing NSCLC cells. (**G**, **H**) EdU assay assessing the impact of AG490 on LCN2-overexpressing NSCLC cells. Data are presented as mean ± SEM (*n* = 3). *P < 0.05; **P < 0.01; ***P < 0.001

To explore the mechanisms by which LCN2 regulates NSCLC progression, we assessed the JAK2/STAT3 signaling pathway, which is implicated in tumor proliferation and metastasis. LCN2 knockdown significantly decreased the phosphorylation of JAK2 and STAT3, while total JAK2 and STAT3 levels remained unchanged. In addition, the level of SOCS3 protein remained unchanged (Fig. 6A & B). Conversely, LCN2 overexpression increased p-JAK2 and p-STAT3 levels (Fig. 6C & D). Collectively, these findings demonstrate that LCN2 activates the JAK2/STAT3 signaling pathway in NSCLC.

**Fig. 7:**
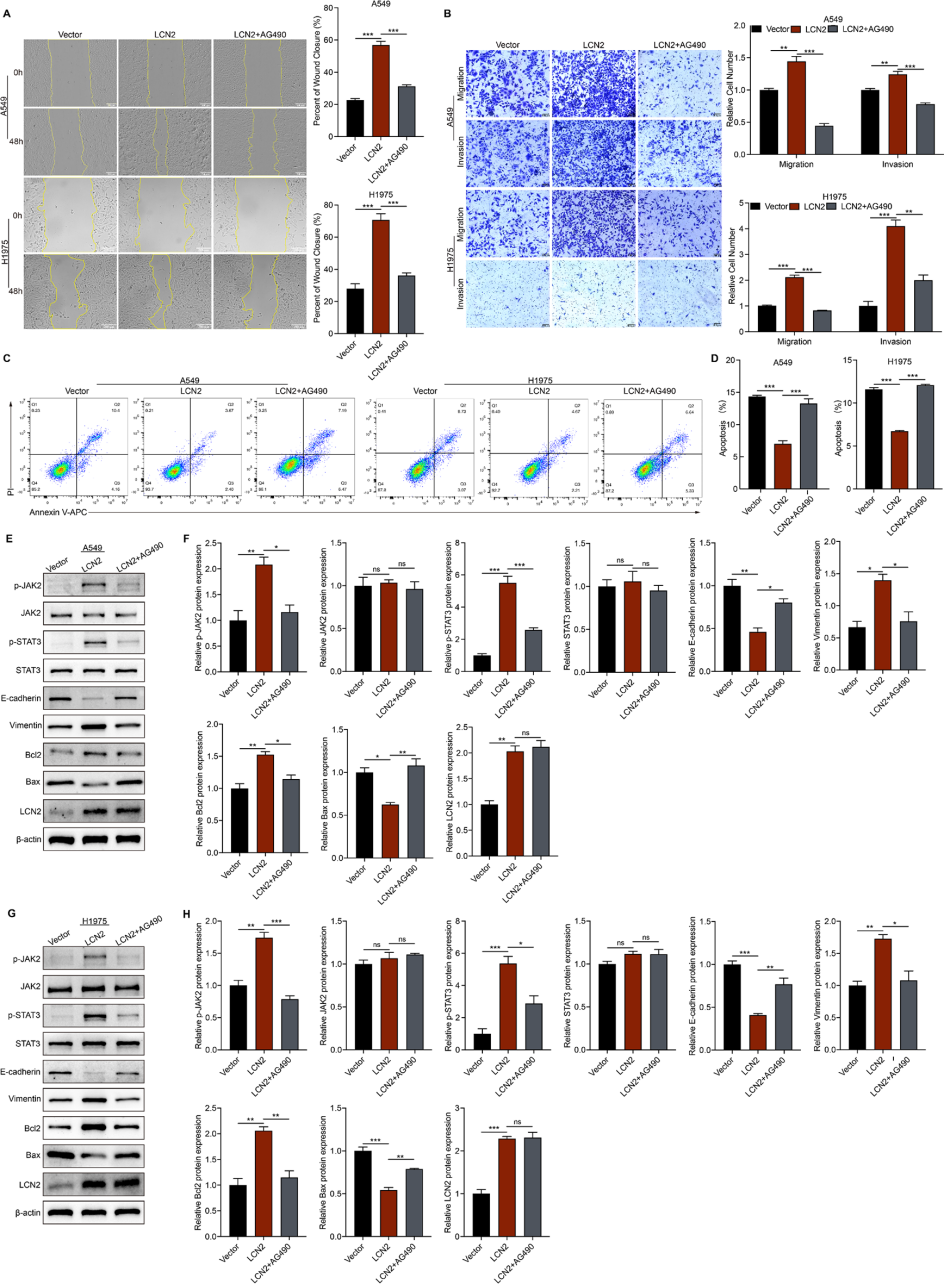
LCN2 promotes NSCLC metastasis and inhibits apoptosis via the JAK2/STAT3 pathway. (**A**) Wound healing assay evaluating the effect of AG490 on the migratory capacity of LCN2-overexpressing NSCLC cells. (**B**) Transwell migration and invasion assays assessing the effects of AG490 on LCN2-overexpressing NSCLC cells. (**C**, **D**) Flow cytometry analysis of apoptosis in LCN2-overexpressing NSCLC cells treated with AG490. (**E**, **F**) Western blot and quantitative analysis of p-JAK2, JAK2, p-STAT3, STAT3, E-cadherin, Vimentin, Bax, Bcl2, and LCN2 expression in A549 cells following AG490 treatment. (**G**, **H**) Western blot and quantitative analysis of p-JAK2, JAK2, p-STAT3, STAT3, E-cadherin, Vimentin, Bax, Bcl2, and LCN2 expression in H1975 cells following AG490 treatment. Data are presented as mean ± SEM (*n* = 3). *P < 0.05; **P < 0.01; ***P < 0.001

Treatment with the JAK2/STAT3 inhibitor AG490 (50 µM) effectively reversed the proliferative effects induced by LCN2 overexpression, as shown by CCK-8 (Fig. 6E & F) and EdU assays (Fig. 6G & H). Similarly, AG490 attenuated LCN2-induced migration and invasion (Fig. 7A & B) and reversed the inhibitory effect of LCN2 on apoptosis (Fig. 7C & D). Western blot analysis confirmed that AG490 specifically inhibited JAK2/STAT3 phosphorylation without altering LCN2 expression and reversed the effects of LCN2 on EMT markers and apoptosis-related proteins (Fig. 7E -H). Collectively, these findings demonstrate that LCN2 promotes NSCLC progression by activating the JAK2/STAT3 signaling pathway.

### LCN2 promotes JAK2/STAT3 activation by interacting with SOCS3

**Fig. 8:**
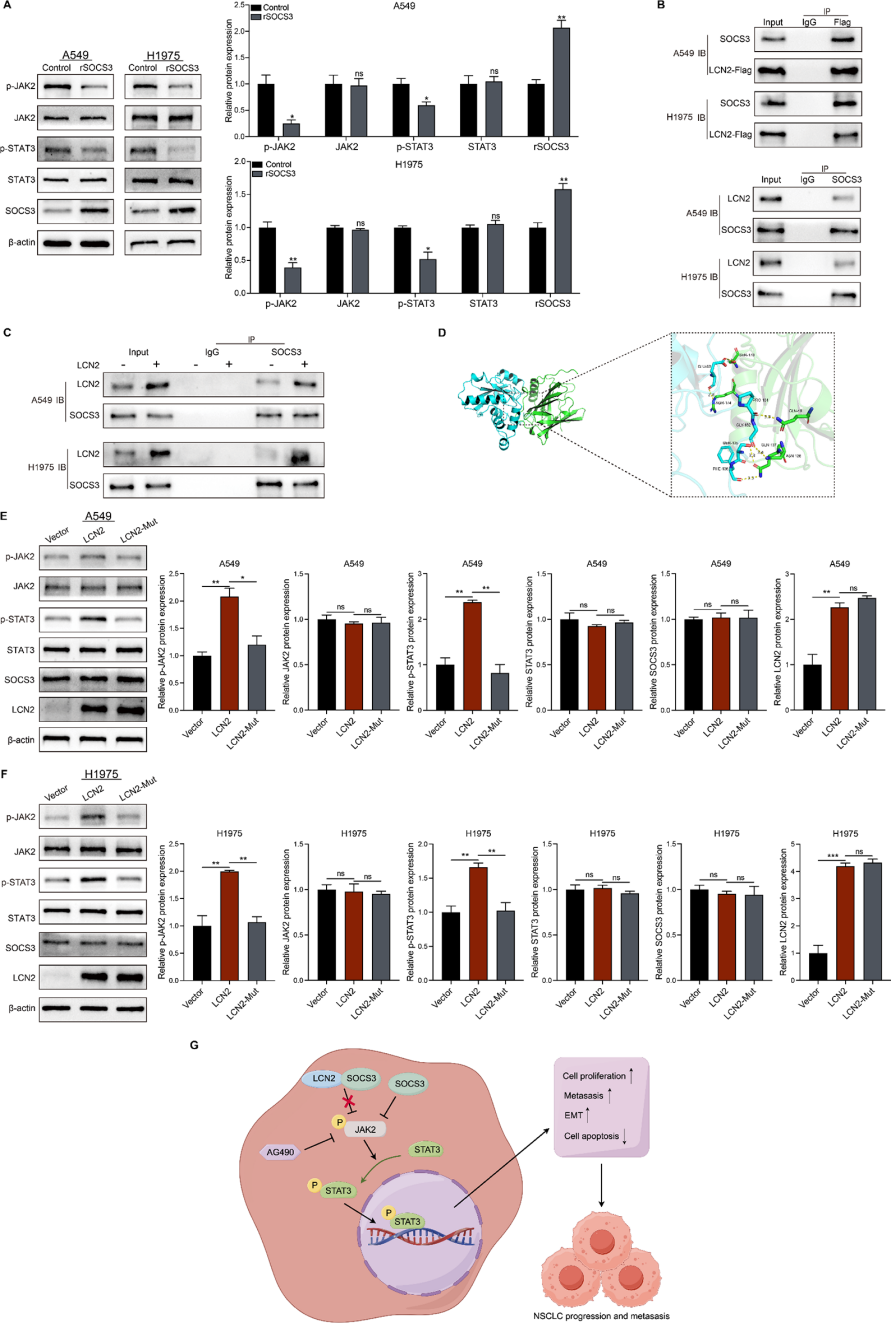
LCN2 promotes JAK2/STAT3 activation by interacting with SOCS3. (**A**) Western blot analysis and quantification of p-JAK2, JAK2, p-STAT3, STAT3, and SOCS3 expression in A549 and H1975 cells treated with recombinant SOCS3. (**B**, **C**) Co-immunoprecipitation and Western blot analysis of LCN2-SOCS3 interaction. (**D**) Molecular docking analysis of LCN2-SOCS3 interaction performed using PyMOL. Green represents the molecular structure of LCN2, while blue represents SOCS3. (**E**, **F**) Western blot and quantification of p-JAK2, JAK2, p-STAT3, STAT3, SOCS3, and LCN2 expression in A549 and H1975 cells transfected with vector, LCN2, or LCN2-Mut plasmids. (**G**) Schematic representation of the proposed oncogenic role of LCN2 in NSCLC through regulation of the JAK2/STAT3 signaling pathway, created using Figdraw. Data are presented as mean ± SEM (*n* = 3). *P < 0.05; **P < 0.01; ***P < 0.001

To further understand how LCN2 regulates the JAK2/STAT3 pathway, NSCLC cells were stimulated with recombinant SOCS3 protein, which inhibited JAK2 and STAT3 phosphorylation, confirming SOCS3 as a negative regulator of this pathway (Fig. 8A). Co-IP assays revealed a potential interaction between LCN2 and SOCS3 (Fig. 8B & C). The interaction between LCN2 and SOCS3 proteins was explored through molecular docking, and the complex with the strongest binding affinity were selected for visualization (Fig. 8D). Mutation of the LCN2-SOCS3 binding site attenuated LCN2-induced phosphorylation of JAK2 and STAT3 (Fig. 8E & F). These results suggest that LCN2 promotes JAK2/STAT3 activation by binding to SOCS3, thereby modulating NSCLC progression.

## Discussion

Early-stage NSCLC patients generally have favorable outcomes, as they are typically eligible for curative surgery and radiotherapy. However, patients with locally advanced or metastatic disease face a high risk of recurrence and poor prognosis [24, 25]. This underscores the urgent need to identify more precise biomarkers and novel therapeutic targets to improve diagnosis and treatment. In this study, we elucidated the oncogenic role of LCN2 in NSCLC progression and explored its underlying molecular mechanisms using comprehensive clinical samples, as well as in vitro and in vivo models (Fig. 8E).

LCN2, a member of the lipocalin family, is a 25 kDa secreted glycoprotein composed of 178 amino acids [26]. Initially characterized by its upregulated expression during infection, LCN2 was originally presumed to primarily regulate immune responses [27]. However, subsequent research has revealed its broad expression across multiple tissues and its association with a spectrum of diseases, most notably cancer [28]. Despite these findings, the role of LCN2 in lung cancer development remains controversial. Some studies have suggested a pro-tumorigenic function for LCN2 in NSCLC. For instance, Song et al. reported that LCN2 downregulation suppresses LUAD growth, potentially through oxidative stress modulation involving the Nrf2/HO-1 pathway [29]. Similarly, Pan et al. demonstrated that LCN2 inhibition enhances ferroptosis, thereby restraining NSCLC progression [30]. Conversely, other studies have proposed that elevated LCN2 levels in early-stage lung cancer may serve as a protective host response, potentially mitigating pro-tumor inflammation. Moreover, LCN2 has been implicated in counteracting LUAD development associated with chronic obstructive pulmonary disease (COPD) by enhancing anti-tumor immune responses [31]. Our findings demonstrate that LCN2 promotes NSCLC proliferation and metastasis, supporting its pro-tumorigenic role in lung cancer. This discrepancy may stem from our study’s focus on NSCLC tumorigenesis, which did not account for the potential involvement of LCN2 in the inflammatory immune processes of tumor development. Future studies should investigate the interplay between LCN2 and the immune microenvironment to achieve a more comprehensive understanding of its role in NSCLC pathogenesis.

The JAK2/STAT3 signaling pathway, driven by tyrosine kinase-associated receptors, plays a crucial role in various biological processes, including hematopoiesis, immune regulation, and embryonic development. Additionally, this pathway is closely linked to key oncogenic processes such as tumor cell proliferation, angiogenesis, and metastasis [32–34]. Emerging evidence highlights the critical role of JAK2/STAT3 signaling in lung cancer progression. For instance, FXR has been shown to promote NSCLC metastasis by transcriptionally upregulating IL-6ST and IL-6, thereby activating the JAK2/STAT3 cascade [18]. Conversely, SH2B3 has been identified as an inhibitor of NSCLC progression, suppressing JAK2/STAT3 signaling to induce apoptosis and reduce EMT, thereby attenuating proliferation, migration, and invasion [35]. However, the functional relationship between LCN2 and the JAK2/STAT3 pathway in NSCLC remains poorly understood. In this study, we observed that modulation of LCN2 expression influenced p-JAK2 and p-STAT3 protein levels, while total JAK2 and STAT3 protein levels remained unchanged. Furthermore, treatment with the JAK2/STAT3 inhibitor AG490 significantly reversed LCN2-induced NSCLC cell proliferation, migration, and invasion. These findings suggest that LCN2 may exert its oncogenic effects in NSCLC through activation of the JAK2/STAT3 signaling pathway, highlighting a potential therapeutic target for intervention.

SOCS3 serves as a crucial negative regulator of the JAK2/STAT3 signaling pathway, playing a pivotal role in its suppression [36]. By suppressing JAK2 activity, SOCS3 effectively hinders tumor cell proliferation, migration, invasion, and tumorigenicity while promoting apoptosis [37, 38]. Specifically, SOCS3 blocks signaling by directly inhibiting JAK enzyme activity [39]. Our study revealed that LCN2 protein interacts with SOCS3 protein, and disrupting this interaction effectively blocked LCN2-mediated activation of the JAK2/STAT3 pathway. These findings establish SOCS3 as a critical regulator of the LCN2/JAK2/STAT3 axis, further underscoring its potential as a therapeutic target in NSCLC.

## Conclusions

This study is the first to identify LCN2 as an oncogene that promotes NSCLC proliferation and metastasis by activating the JAK2/STAT3 signaling pathway through interaction with SOCS3. Our findings provide new insights into the molecular mechanisms underlying NSCLC progression and offer a foundation for future studies exploring the intricate interplay between LCN2 and the JAK2/STAT3 pathway. In summary, these results suggest that LCN2 represents a promising therapeutic target for NSCLC, warranting further investigation into its clinical applicability.

## Electronic supplementary material

Below is the link to the electronic supplementary material.

Supplementary Material 1

Supplementary Material 2

## Data Availability

The publicly available data used in the present study can be found in the UALCAN (https://ualcan.path.uab.edu), TCGA (https://portal.gdc.cancer.gov), UCSC-XENA (https://xena.ucsc.edu), GEO (https://www.ncbi.nlm.nih.gov/geo/), GuangRe biotechnology (https://grswsci.top), and KM survival analysis (https://kmplot.com) databases. The other data generated in the present study are available from the corresponding author on reasonable request.

## Abbreviations

CCK-8: Counting Kit-8
Co-IP: Co-Immunoprecipitation
EdU: 5-Ethynyl-2′-deoxyuridine
EMT: Epithelial-Mesenchymal Transition
H&E: Hematoxylin–eosin staining
IHC: Immunohistochemistry
JAK2: Janus Kinase 2
KM: Kaplan-Meier
LCN2: Lipocalin-2
LUAD: Lung Adenocarcinoma
LUSC: Lung Squamous Carcinoma
NSCLC: Non-Small Cell Lung Cancer
OS: Overall Survival
qRT‒PCR: Quantitative Real-Time Polymerase Chain Reaction
shRNA: Small Hairpin RNA
STAT3: Signal Transducer and Activator of Transcription 3
TCGA: The Cancer Genome Atlas
UALCAN: The Universal Analysis of Cancer
GEO: Gene Expression Omnibus
