# Supplementary figures and images for: Lipocalin-2 promotes NSCLC progression by activating the JAK2/STAT3 signaling pathway
Source: J Transl Med. 2025 Apr 10;23:419. doi: 10.1186/s12967-025-06418-1 (PMC11987316; doi:10.1186/s12967-025-06418-1)

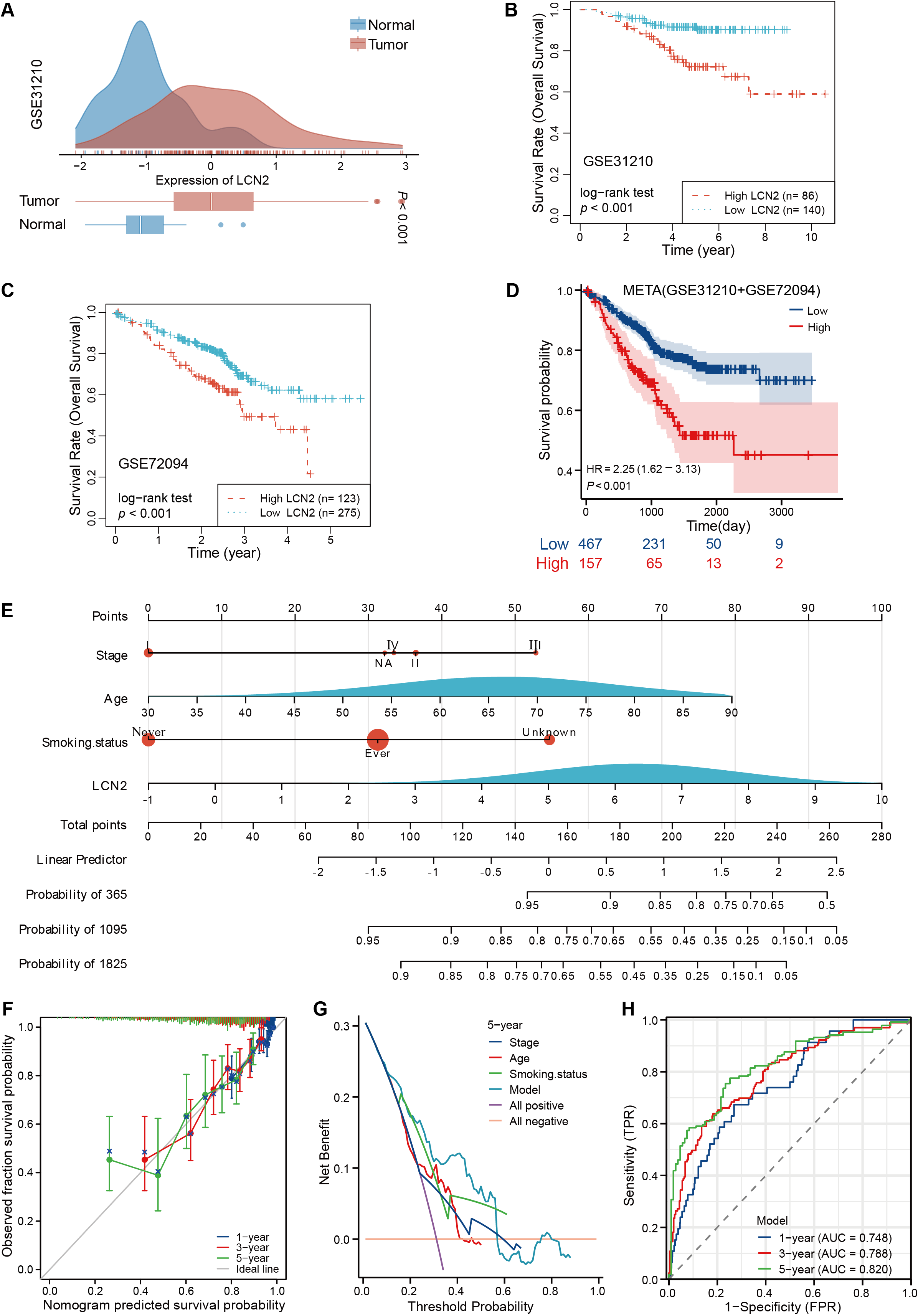

Supplement: Supplementary file 1 — Supplementary Material 1 [file 12967_2025_6418_MOESM1_ESM.png]
