# Supplementary material for: Lipocalin-2 promotes NSCLC progression by activating the JAK2/STAT3 signaling pathway
Source: J Transl Med. 2025 Apr 10;23:419. doi: 10.1186/s12967-025-06418-1 (PMC11987316; doi:10.1186/s12967-025-06418-1)
Supplement: Supplementary file 2 — Supplementary Material 2 [file 12967_2025_6418_MOESM2_ESM.docx]

**Supplement Figure legends**

Supplementary Figure 1. (A) LCN2 expression levels in normal and tumor samples from the GSE31210 dataset. (B) Kaplan-Meier survival curve for OS based on LCN2 expression in the GSE31210 dataset. (C) Kaplan-Meier survival curve for OS stratified by LCN2 expression in the GSE72094 dataset. (D) Kaplan-Meier survival curve for OS based on LCN2 expression in the META cohort (combined GSE31210 and GSE72094 datasets). (E) Prognostic nomogram predicting patient survival in the META cohort. (F) Calibration plots assessing the accuracy of the nomogram-predicted 1-, 3-, and 5-year OS in the META cohort. (G) Decision curve analysis evaluating the clinical utility of the nomogram for predicting 5-year survival in the META cohort. (H) Time-dependent ROC curve analysis assessing the predictive performance of the nomogram for survival in the META cohort.
